# Assessment of Effect of Perceived Social Support on School Readiness, Mental Wellbeing, and Self-Esteem: Mediating Role of Psychological Resilience

**DOI:** 10.3389/fpsyg.2022.911841

**Published:** 2022-05-30

**Authors:** Yefei Shi

**Affiliations:** College of Fine Arts, Tangshan Normal University, Tangshan, China

**Keywords:** perceived social support (PSS), mental wellbeing (MWB), left behind children, psychological resilience (PR), school readiness (SR), self-esteem (SE)

## Abstract

Objective of this study is to investigate the impact of perceived social support on the self-esteem, mental wellbeing, and school readiness of left-behind (LB) children. It further aims at understanding the mediating role of psychological resilience between the relationships of perceived social support and self-esteem, mental wellbeing, and school readiness. For this purpose, population frame of the LB children between the ages of 8–12 years in Mainland China was taken. The sample size of 335 was taken to reach the findings through partial least square (PLS) structural equation modeling. The SmartPLS has been used to analyze the data. The results obtained in this study have shown that the perceived social support plays a very significant role in enhancing the mental wellbeing, self-esteem, and school readiness of the LB children. It has also been found that the perceived social support plays a positive role in the psychological resilience. Furthermore, it has also been found that the psychological resilience is an important predictor of self-esteem and school readiness. Further, the psychological resilience has proved to be significant mediator between the relationship of the perceived social support and self-esteem; and also between the relationship of the perceived social support and school readiness.

## Introduction

The policymakers and researchers have given vast attention to the left-behind (LB) children (Wang et al., 2019). The policymakers have been finding ways to help the LB children because the psychological health of these children is at stake (Ran et al., 2022). Moreover, various researchers (Fan and Lu, 2020; Chen et al., 2021) have carried out the studies to understand the factors that affect the LB children. The children who are under 18 and are LB by their parents for more than 6 months are defined LB children. The parents of these children usually leave their children behind their original residence and migrate somewhere else for work (Wen et al., 2021). In China and other countries, an increasing trend of LB children has been observed (Gu, 2021). Particularly, in Thailand, 20% of children were left behind by their parents as these parents migrated to other areas for earning a living. Based on the recent statistics of China, there were ~55 million LB children, and 48.09% were left apart from their parents (Tan and Ma, 2021). Therefore, the LB children are increasing at a fast rate.

The reason for the increase in the LB children is the on-going development of urban areas and the difference in the social system of urban and rural areas which provide various opportunities for people to move to the urban regions of better income (Li et al., 2021). Due to this reason, the parents get separated from their children and the children stay in their residence (Li et al., 2021). These children lack emotional support as they stay away from their parents. As a result, the children suffer in terms of psychological health. Studies have demonstrated that the psychological health of the children is negatively impacted by a lack of parental care (Dai and Chu, 2018). Some of the most common psychological problems observed among LB children are anxiety, depression, loneliness, and self-esteem. Recently, the researchers pay great attention to the self-esteem of the LB children. Self-esteem is referred to as the attitude toward the self which could be negative or positive and is generally used to describe the self-worth (Rosenberg et al., 2018). The LB children have low self-esteem as “left behind” is considered a negative life event. According to Lan et al. (2019), strong ties of children with their parents develop high self-esteem among children, while long-term parental separation hampers their self-esteem.

The LB children have a quite weakened relationship with their parents; as a result, the mental wellbeing of such children is poor with poor academic performance (Lei et al., 2019a). The separation of LB children from their parents lacks the communication skills of these children; therefore, they find it difficult to communicate with their teachers and classmates (Fellmeth et al., 2018). The LB children become the victim of loneliness which deteriorates their mental wellbeing because of a lack of family support. Additionally, these LB children see their friends or classmates, who are living along with their parents, creates depression and hampers their mental health and wellbeing (Tan et al., 2020). Moreover, the LB children in China are vulnerable to depression and this depression, in turn, causes the mental problem and lower mental wellbeing among these children. Furthermore, the academic performance of LB children is low as compared to the children who live with their parents; this further causes low mental wellbeing among the LB children (Man and Cao, 2020). This indicates that the LB children have low mental wellbeing because they stay away from their parents.

The LB children face various social factors that influence the high risk of low school readiness among such children. This risk is apparent before entering school and continues throughout school life (Hu et al., 2020). Additionally, the risk associated with the separation of children from their parents is poor school readiness along with low academic performance (Li et al., 2021). The LB children are deprived of the love and care from their parents, which disengages them from entering to school. Moreover, such children hesitate to communicate with the teachers and classmates; therefore, they avoid entering to school and thus low school readiness has been found among them (Gan et al., 2016). Also, loneliness among these children makes them introverted; therefore, they show reluctant to attend school.

Social support is a crucial social factor that helps in child development and improves the overall mental wellbeing of children (Lei et al., 2019a). Social support is referred to as a feeling of an individual that the society shows love and care and the individual is part of the social network (Cohen and Wills, 1985). The LB children perceive social support as a significant and positive indicator that helps them improve their overall academic performance and bonds them with the society. The social network provides supportive assistance to the LB children which often leads toward a better mental health and wellbeing of the children. Additionally, social support enhances the life satisfaction and psychological wellbeing of the LB children because such children acquire love and care from the society (Cui et al., 2021). Although LB children experience a high level of psychological distress, social support remains as a ray of hope for them.

Another factor that positively affects the psychological wellbeing of the LB children is psychological resilience. Psychological resilience refers to the capacity of an individual to adapt to the disturbances and quickly return to pre-crisis status (Masten, 2014). Resilience strengthens the ability of an individual to contribute to good developmental outcomes (Li et al., 2021). The LB children who have high resilience possess self-efficient, social competence, autonomy, and sense of purpose (Xiao et al., 2019). Such children have the ability to respond to the dilemmas and risks. Studies showed and presented that the children with high resilience have better mental wellbeing as opposed to the children with low resilience. Psychological resilience among LB children is beneficial in achieving improved psychological status even in hostile situations (Ran et al., 2022).

A study conducted by Fan and Lu (2020) examined the role of perceived social support on mental wellbeing with the mediation of resilience of the LB children. The author of this study suggested inculcating other factors that could impact the relationship between those study variables. Therefore, to enrich the framework and mechanism of the existing model, two new variables, i.e., self-esteem and school readiness have been investigated. Moreover, a lack of studies has analyzed the role of school readiness among LB children; therefore, this relationship of perceived social support and school readiness and also the relationship of psychological resilience and school readiness have been examined. Additionally, Zhao et al. (2020a) also suggested incorporating other comprehensive measures that could affect the LB children; therefore, this study aimed to effect perceived social support on school readiness, mental wellbeing, and self-esteem with the mediation of psychological resilience among the Chinese LB children.

The gap in the literature would be filled by addressing the research objectives of the study. The research objectives of the study are as follows: To (1) examine the impact of perceived social support on mental wellbeing, (2) determine the influence of perceived social support on self-esteem, (3) assess the impact of psychological social support on school readiness, (4) examine the role of perceived social support on psychological resilience, (5) determine the influence of psychological resilience on mental-wellbeing, (6) analyze the effect of psychological resilience on self-esteem, and (7) examine the impact of psychological resilience on school readiness. The study also developed some objectives related to the mediating mechanism of psychological resilience, and those objectives are listed as follows: To (1) analyze the mediating mechanism of psychological resilience in the relationship between perceived social support and mental wellbeing, (2) assess the mediating role of psychological resilience in the relationship between perceived social support and self-esteem, and (3) examine the mediating role of psychological resilience in the relationship between perceived social support and school readiness. Therefore, the study aims to investigate the relationship between these constructs.

## Review Of Literature and Hypotheses Development

### Self-Enhancement Theory

Self-enhancement theory suggests that motivation is developed among individuals in making people have a good perception about him and established high self-esteem (Sedikides and Gregg, 2008). This motive is highlighted in case of failure, threat, or blows to one's self-esteem. This theory focuses on positive aspects over negative self-views. Self-enhancement is one of the four self-evaluation motives with self-assessment, self-improvement, and self-regulation. Self-evaluation motive focuses on the process of self-regulation and is mainly done to improve oneself. Self-assessment drives an accurate self-concept, and self-improvement focuses on getting better with time. These aspects motivate the individuals to improve their overall mental wellbeing as well as self-esteem. Moreover, self-enhancement theory can be applied in different contexts and under different situations. The explanation of self-enhancement can be done in different ways, depending on the nature of the context.

Self-enhancement theory has been widely used in social psychology literature. Dufner et al. carried out a study to examine the impact of self-enhancement on personal adjustment and interpersonal adjustment using self-enhancement theory. This theory supported the findings that self-enhancement is positively related to both personal and interpersonal adjustment. Another study that incorporated this theory is conducted by Grover to examine the effect of respect on performance with the moderation of self-esteem. The study presented that this theory positively influences the behavior of the people such that self-esteem strengthens the relationship between the performance and the work efforts by the individuals.

This study has incorporated self-enhancement theory as it states that individuals develop high self-esteem, especially in situations of threat or failure. The current study focuses on the LB children who are deprived of the love and care of their parents as the parents stay away from their children. The children feel depressed and anxious as they are separated from their parents for a longer period. This situation is quite threatening and it reduces self-esteem, reduces mental wellbeing, and lowers school readiness among the LB children. In the light of this theory, perceived social support and psychological resilience are the two motivators that can make people feel good and enhance their overall self-esteem, mental wellbeing, and school readiness.

### Relationship Between Perceived Social Support and Self-Esteem

Perceived social support is the evaluation and expectation of an individual for social support and a belief that they will receive social support (Feng et al., 2018). Social support is necessary for situations when a person is facing difficulties. Therefore, when people receive social support, their self-esteem rises and they feel satisfied (Wang and Xie, 2020). In the case of the LB children, they desperately need social support because they are deprived of their parental care. Social support helps to develop self-esteem among them (Ren and Li, 2020). Protective factors such as perceived social support and psychological safety may help the LB children buffer against the negative situations and enhances self-esteem (Cui et al., 2021). Perceived social support is imperative for the LB children because this factor allows them to interact and socialize with their peers and as a result, they develop self-esteem.

According to Gu (2021), perceived social support is closely related to self-esteem. Individuals having extremely low social support would have low satisfaction levels which would ultimately result in low self-esteem (Xin et al., 2019). Studies have shown that the individuals with high social support tend to have higher levels of self-esteem (Fellmeth et al., 2018). Liu et al. (2019) also support these findings and suggested that social support has a direct relationship with self-esteem. According to socio-meter theory, self-esteem reflects social connection and fosters positive social support which produces higher self-esteem (Li et al., 2018). Social support enables the LB individuals to socialize with others which enhances the self-esteem of these children (Tan et al., 2022). A study conducted by Jin and Zhu (2022) examined how perceived social support is related to self-esteem, and showed that these constructs have a positive relationship. Another recent study by Ren and Li (2020) investigated the impact of perceived social support and self-esteem among the LB children. The study revealed that the LB children feel satisfied by social support which increases their self-esteem. Many studies have not explored the relationship between perceived social support and self-esteem; therefore, there is room to investigate this relation. Thus, the Hypothesis H1 is proposed as follows:

**H1**. Perceived social support has an effect on self-esteem.

### Relationship Between Perceived Social Support and Mental Wellbeing

In the child development process, the role of social support is significant as it helps to improve the mental wellbeing of the children (Zhang et al., 2019). Social support develops a feeling of love and cares among children and they perceive to be part of the social supportive network (Man and Cao, 2020). Social support can be either actual or perceived. However, perceived social support has a stronger association with the mental wellbeing of the LB children as compared to actual social support (Chen et al., 2021). Social support provides assistance to people; therefore, social support tends to improve the mental wellbeing of individuals (Wang and Liu, 2021). Moreover, social support also enhances the life satisfaction of children which results in better psychological wellbeing. Additionally, social support contributes to a higher level of mental wellbeing regardless of stress and pressure (Wang et al., 2021). Social support allows the children to communicate and share problems with others in the society which leads to the better mental wellbeing of children.

The positive social interaction among peers and friends develops interpersonal relationships which helps to release tension and anxiety of children; thus improving the psychological wellbeing of the LB children (Lei et al., 2019a). Social support enables the LB children to share their feelings, ideas, and thoughts with others. For example, if the LB children want assistance, they can talk to others which improves their mental wellbeing (Wang and Liu, 2021). Moreover, teachers can assess the student's academic performance and provide reasonable suggestions which would help solve psychological problems and promote the mental wellbeing of students (Wang and Liu, 2021). Studies have shown that perceived social support is positively associated with the mental wellbeing of LB children because these children need attention and care from others (Wang and Xie, 2020). There is a pressing need to investigate the relationship between perceived social support and mental wellbeing; thus, this study aimed to analyze the perceived social support on the mental health of the LB children. Our Hypothesis H2 is proposed as follows:

**H2**. Perceived social support has an effect on mental wellbeing.

### Relationship Between Perceived Social Support and School Readiness

Perceived social support is positively associated with outcomes such as improved wellbeing, better interpersonal skills, and self-management (Hu et al., 2020). Studies suggest that the social support is a positive indicator for individuals and people who received social support tend to exhibit positive outcomes (List et al., 2021). From the perspective of the developmental systems, school readiness considers the competencies of children as the outcome of interest and emphasizes the social system within which the children are embedded (Chen et al., 2021). These social supports thereafter support or inhibit school readiness among the children. Upon this argument, researchers built that the LB children are grown without parental care; therefore, perceived social support is a significant factor that encourages school readiness among them (Wen et al., 2021). School readiness is developed when the child is mentally prepared to enter school, and it becomes quite challenging for the child to engage in school readiness without their parents (Mathis et al., 2022). Thus, the role of perceived social support is important in influencing school readiness among children.

Developing school readiness is an important factor for the LB children because these children are deprived of parental care and love. It becomes crucial to develop to investigate the factor that can influence and hamper school readiness among these children. One such factor is perceived social support as this factor helps in exhibiting positive outcomes among children (Gan et al., 2016). In addition, perceived social support helps the LB children to cope up with school matters; therefore, perceived social support fosters in developing school readiness among such children (Wen et al., 2021). The direct relationship between perceived social support and school readiness has not been investigated and a lack of studies has been conducted in this regard. Therefore, it becomes significant to examine the association between perceived social support and school readiness among the LB children. Therefore, the Hypothesis H3 has been proposed as follows:

**H3**. Perceived social support has an effect on school readiness.

### Relationship Between Perceived Social Support and Psychological Resilience

Perceived social support has a positive association with stress regulation as socializing helps individuals to communicate and relieve stress and trauma (Huang et al., 2020). High social support is significant in promoting children with abnormalities. Moreover, high social support increases self-confidence, decreases the likelihood of engaging in risky behavior, and fosters effective coping strategies (e.g., psychological resilience) (Ran et al., 2022). Perceived social support also encourages healthy coping behavior and enhances emotional regulations such as anxiety, depression mistrust, and fear (Dai and Chu, 2018). Consequently, these factors contribute to the development of psychological resilience and help to promote a better lifestyle for individuals. Psychological resilience is important in overcoming the challenges faced by the LB children (Li et al., 2018). Such children already face many difficulties in life which make them anxious, depressed, and lonely; thus, perceived social support and psychological resilience are important for them.

Strategies must be devised for the LB children to cope with stress and trauma because such children might indulge in deviant behavior which would further worsen the situation (Parviniannasab et al., 2022). However, with perceived social support and psychological resilience, children can better come with adversity, other negative life events, and mental health problems (Chai et al., 2019). Findings from the previous studies showed that psychological resilience is a promising factor that can reduce stress and chronic stressors among the LB children (Zhao et al., 2018). On the contrary, low psychological resilience has been associated with depression which means that psychological resilience is positively associated with improved mental health (Ran et al., 2022). The social support received by children helps them to develop psychological resilience. Few studies have been conducted to examine the association between perceived social support and psychological resilience; therefore, there is still a need to explore this relationship in-depth. Thus, Hypothesis H4 has been established as follows:

**H4**. Perceived social support has an effect on psychological resilience.

### Relationship Between Psychological Resilience and Self-Esteem

Psychological resilience is the main component of the risk-protective model which promotes factors like a supportive system and positive personal traits to minimize risks and negative outcomes (Man and Cao, 2020). According to Feng et al. (2018), various risks are associated with the LB children such as psychological problems, depression, and even suicidal intentions. These risks can be mitigated if the LB children possess psychological resilience because resilience can subsequently help to overcome such risks through positive self-esteem (Tan et al., 2020). Zhao et al. (2020b) found that psychological resilience affects both self-esteem and depression among the LB children. Moreover, the self-esteem of the LB children can be improved if such children develop psychological resilience so that they can withstand challenging situations and improve their academic performance (Fellmeth et al., 2018). A resilient individual knows his capabilities and he feels good about himself; thus, increasing his level of self-esteem (Yu et al., 2022). Psychological resilience is necessary among LB children because they already feel neglected; however, the power of resilience can boost their self-esteem (Gabrielli et al., 2022).

The level of self-esteem is enhanced as a result of positive psychological factors such as psychological resilience (Zhao et al., 2020a). Psychological resilience reduces depression and anxiety among the LB children while improving their self-esteem and self-efficacy (Yu et al., 2022). For example (Gabrielli et al., 2022), conducted a study on the psychological resilience of the LB children and found that psychological resilience increases self-esteem and reduced depression. The development level of the LB children's emotional performance, health, and psychological cognition got affected when their parents left home. Thus, the need for some positive psychological factors is crucial for the child's upbringing and better future. Therefore, it becomes imperative to examine the relationship between psychological resilience and self-esteem among the LB children. Summing it up, although the role of psychological resilience has been explored in different contexts, still there is not much literature available on how psychological resilience influences self-esteem among the LB children. Our Hypothesis H5 is proposed as follows:

**H5**. Psychological resilience has an effect on self-esteem.

### Relationship Between Psychological Resilience and Mental Wellbeing

The mental wellbeing of the LB children gets suffered as soon as their parents leave them (Chai et al., 2019). This results in aggressive behavior of such children with depression, anxiety, and loneliness (Gan et al., 2016). However, developing positive psychological factors would be beneficial for the LB children. In this regard, Wang and Xie (2020) investigated the impact of some positive psychological factors that would influence the mental wellbeing of the LB children. The result of the study showed that psychological resilience was found to be a positive indicator of the mental wellbeing of LB children. Moreover, children with higher psychological resilience can actively respond to dilemmas and risks (Lei et al., 2019a). Studies indicated that psychological resilience is positively associated with mental wellbeing. Despite threatening circumstances, the LB children can develop mental wellbeing through psychological resilience because resilience enables them to quickly overcome challenging situations (Cui et al., 2021).

On the contrary, the LB children having low psychological resilience can easily develop symptoms of depression, anxiety, and would feel lonely, which would ultimately result in lower mental wellbeing (Parviniannasab et al., 2022). The LB children who have high resilience tend to have high school readiness because such children can overcome their weaknesses and face challenging situations. Psychological problems hamper the mental wellbeing of individuals as such problems disturb their mental health. For instance, (Tang et al., 2018) examined the impact of psychological problems on the mental health of teenagers using a cross-sectional study design. The results revealed that psychological problems deteriorate the mental health of teenagers from time to time. Moreover, stressful life events and depression are among the most common psychological problems observed in individuals (Chai et al., 2019). Therefore, there is a dire need to investigate the positive psychological factors that would affect the mental wellbeing of people. Although studies have been carried out to examine the role of psychological resilience on mental wellbeing, a lack of evidence is present in the literature about the LB children. Therefore, the study postulated the following Hypothesis H6:

**H6**. Psychological resilience has an effect on mental wellbeing.

### Relationship Between Psychological Resilience and School Readiness

Protective factors such as psychological resilience are significant, particularly, to the understanding of preschool to early elementary age children because these children come across different difficulties in coping with academic pressure (Anderson, 2018). However, early parental behavior and beliefs have been found crucial in determining academic readiness and school readiness (Ramakrishnan and Masten, 2019). Additionally, scholars also agree that the parent-child relationship positively impacts the successful development of children which signifies that the parent-child relationship is important for early childhood years (Zhang et al., 2021). Particularly, the parents having less-conflicting relationships with their children develop positive psychological wellbeing and school readiness. Nonetheless, the LB children who have very weak to no relationship with their parents cannot develop school readiness as they are psychologically empowered (Fan and Lu, 2020). Furthermore, the resilient children are capable of withstanding problems which enable the children to develop school readiness. According to Zhou et al. (2020), psychological factors positively influence the LB children because they already feel deprived, depressed, and lonely (Gabrielli et al., 2022) which claimed that psychological resilience is a positive factor that helps the LB children to cope up with adverse situations; therefore, it becomes significant to develop perceived social support to establish psychological resilience in such children.

Psychological resilience brings about various advantages for the LB children; for example, improved psychological wellbeing, empowerment, self-esteem, etc. (Sun et al., 2021). The LB children who have high resilience tend to have high school readiness because such children can overcome their weaknesses and face challenging situations (Chen et al., 2021). Moreover, the LB children require psychological resilience to enter school because the mental wellbeing of these children is affected by the circumstances (Yeung and Li, 2019). The LB children who have high resilience possess self-efficient, social competence, autonomy, and sense of purpose (Gu, 2021). The researchers found that the LB children are reluctant to enter school because of a lack of psychological wellbeing (Ramakrishnan and Masten, 2019). Limited literature is available to explain the relationship between psychological resilience and school readiness among the LB children. Also, it would be interesting to understand how psychological resilience affects school readiness among such children because school is a crucial part of one's upbringing. Hence, this study aimed to examine the relationship between psychological resilience and school readiness; thus, Hypothesis H7 has been formulated as follows:

**H7**. Psychological resilience has an effect on school readiness.

### Mediating Role of Psychological Resilience

Psychological resilience is referred to as the ability of an individual adapt to the disturbances that tend to threaten the function and development of the individuals (Masten, 2014). Psychological resilience enables individuals to develop strength and ability to reduce or minimize risks and contribute to the good development of individuals (Ran et al., 2022). Children with high resilience have characteristics of self-efficacy, autonomy, social competence, sense of purpose, and adaptability (Gabrielli et al., 2022). Such children have the ability to overcome dilemmas and risks. Studies have been carried out to investigate the impact of resilience on the mental wellbeing of children (Anyan et al., 2017). Specifically for the LB children, despite threatening circumstances, such children develop positive psychological wellbeing through resilience. Also, resilience among such children allows them to cope successfully with the LB circumstances and reduce the negative impact of situations resulting from a lack of social support (Zhou et al., 2020).

Resilience as a coping mechanism is related to protective and risk factors (Kumar et al., 2022). Protective factors enable individuals to overcome stressful life events and risks, and risk factors can pose to be harmful to individuals. The enhancement of protective factors positively impacts resilience among children (Wen et al., 2021). Additionally, internal protective factors as well as external protective factors include school, family, and community which play a significant role in developing the resilience of the LB children (Zhang et al., 2021). Resilience benefits the LB children to combat stressful life events and the developmental wellbeing and self-esteem of these children. The mediating role of psychological resilience has been studied by Molla et al. to examine the relationship between social support and spiritual wellbeing with the mediation of psychological resilience among female cancer patients. The study found that social support has a positive relationship with spiritual wellbeing and psychological resilience facilities this relationship. Likewise, Xiang et al. also carried out a study to analyze the impact of envy on depression with the mediation of psychological resilience and social support. The results revealed that social support mediated the relationship between envy and psychological resilience, while psychological resilience mediated the relationship between social support and depression among Chinese college students.

The family bond is one of the most crucial factors that develop the resilience of children. The LB children are separated from their parents therefore their psychological capabilities are hampered. Moreover, the family environment significantly impacts the development of children, and family social support helps in supporting the children (Wen et al., 2021). Therefore, a supportive environment helps the children to develop and improve psychological resilience (Lei et al., 2019b). Developing resilience among the LB children is crucial because such children are deprived of their basic rights and developing resilience can help them to overcome the challenges and improve their overall wellbeing. Social support from friends and peers can help improve the psychological resilience of children (Tan et al., 2020). According to Gu (2021), a good relationship among friends encourages resilience in children. Also, different types of social support such as neighbor support and teacher social support can positively impact the enhancement of resilience of children (Chen et al., 2021). The social networks enable the children to overcome stressful events and thus cultivating the abilities of mental wellbeing. Although, the mediating role of psychological resilience to some extent has been investigated in the relationship between perceived social support, self-esteem, and mental wellbeing, the mediating role of psychological resilience in the relationship between perceived social support and school readiness has not been explored before. Thus, Hypotheses H8–H10 have been developed as follows:

**H8**. Psychological resilience mediates the relationship between perceived social support and self-esteem.
**H9**. Psychological resilience mediates the relationship between perceived social support and mental wellbeing.
**H10**. Psychological resilience mediates the relationship between perceived social support and school readiness.

**Figure 1:**
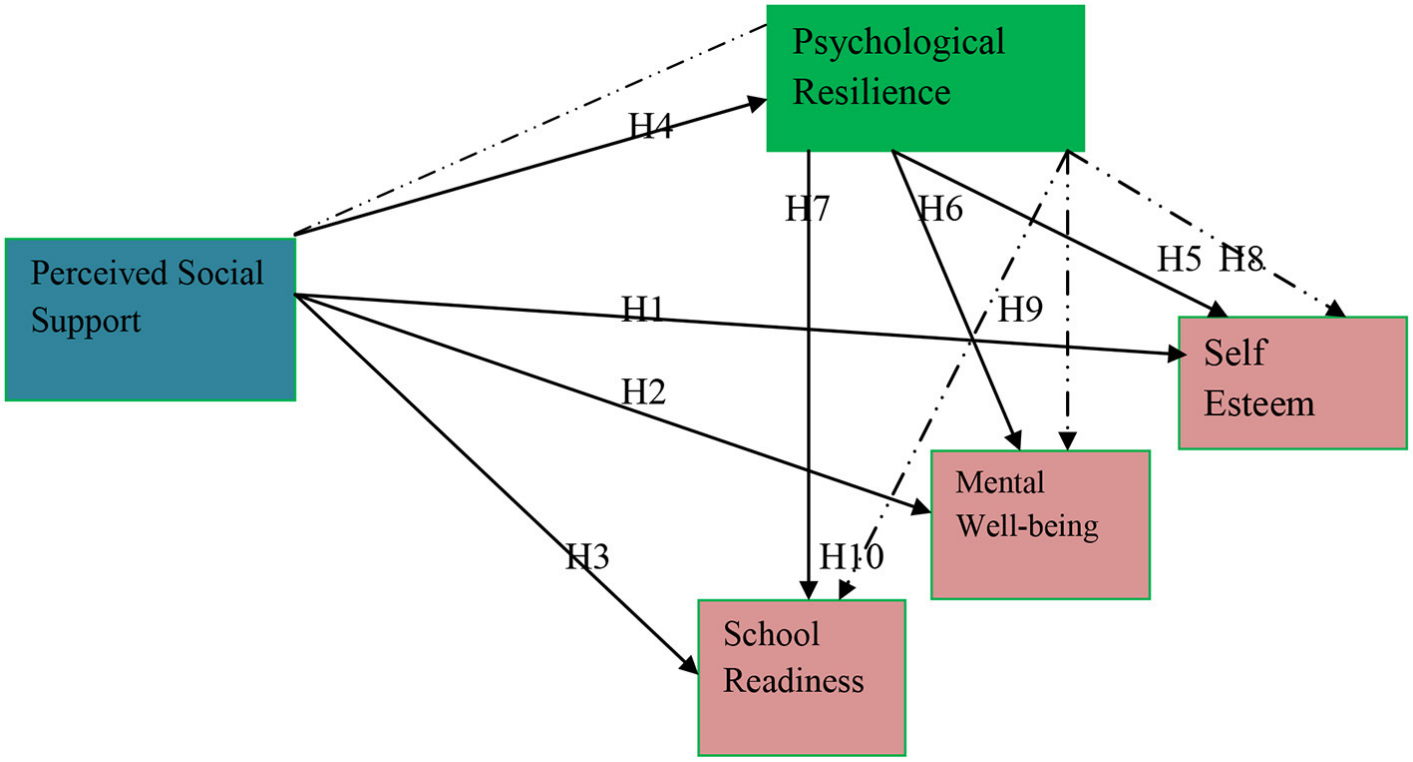
Conceptual framework.

A conceptual model (Figure 1) has been formed based on the above literature and the hypotheses.

## Methodology

For the validation of the hypotheses presented in this study, a quantitative research design has been employed with deductive research approach. The hypotheses help to examine the effect of independent variables on the consequent variables. This research design has been used to avoid the possible biases. The data was collected through self-administered surveys. The target population of the study was the LB children aged from 8 years to 12 years from the schools situated in Mainland China. The items of the questionnaire were kept short and clear to ensure the rationality of data. In this study, the data had been selected on the basis of convenience sampling because not all students' parents were ready to let their children part of the survey (Fan and Lu, 2020). Convenience sampling lets the researcher collect the data from the respondents who are conveniently and readily available. Ethical perspective of the research was kept intact by asking for prior permission from the children's parents. Few parents refused to let their children be part of survey. The sample size of the study was 335; however, 400 questionnaires were distributed; 65 questionnaires had been filled in a way that could not be used for the research; therefore, they were discarded. The questionnaires had been distributed among the students who met the criteria of the LB in this study. They were given a thorough orientation about the protocol and procedure to fill the questionnaires. They were also ensured about there are not absolute right or wrong answer the questions given in the questionnaire. They were given the time of 1 week that questionnaires would be collected a week later. When a week later, the researcher went to collect the questionnaires, approximately one-third of them were unfilled, who were given even a week's more time. Then a week later, they were collected; however, some of the questionnaires were blank that inform us that their parents did not let them fill out the questionnaire. The anonymity of the respondents had been ensured. The unit of analysis for this study was the LB children aged 8–12 years. In this cross-sectional study, the data was collected in 4 weeks making sure haste makes no misunderstanding or spurious responses.

### Statistical Tool

This study employees the mainstream analytical technique of partial least square (PLS) structural equation modeling using the software SmartPLS. This software helps in getting the deep insights despite small sample sizes (Bari et al., 2019). It further allows to develop such path models instantly that can measure the relationships among different variables simultaneously (Sarstedt et al., 2014). Using this software, the data is usually analyzed in two stages. The first stage is named as measurement model and the second stage is named as structural model. Measurement model screens the data for initial validation while the structural model tests the hypotheses.

### Measurement

The questionnaire used in this study has been formatted at 5-point Likert scale. It ranged from 1 to 5 (1 = strongly disagree, 2 = disagree, 3 = neutral, 4 = agree, and 5 = strongly agree). The questionnaire consisted of 29 items in total comprising five subscales. The first scale measured the mental wellbeing consisting of five items adapted from Chopik et al. (2019). The second scale measured the psychological resilience consisting of four items adapted from Zhao et al. (2020a). The third subscale measured the perceived social support consisting of four items adapted from Fan and Lu (2020). The next subscale measured the variable self-esteem consisting of 10 items adapted from Zhao et al. (2020a). The subscale of school readiness was measured with 6-items scale adapted from Hughes et al. (2015). The last part of the questionnaire addressed the information of the respondents regarding gender, number of siblings, and the marital status of parents.

### Demographic Details

The demographic profile of 335 respondents has been analyzed using the gender, grade, marital status of the parents and the siblings. The findings of the study show that there were 64.19% students were male showing dominancy in the sample. The highest number of students was from grade 3 showing 51.94% representation. Further, the highest number of the parents was found divorced showing 47.46% followed by single parents showing 25.67%. While almost half of the respondents had siblings while half had no siblings. The results can be seen in Table 1.

**Table 1:** Demographic analysis.

**Demographics**	**Frequency**	**%**
**Gender**		
Male	166	64.19
Female	169	35.81
**Grade**		
Grade 1	64	19.10
Grade 2	97	28.95
Grade 3	174	51.94
**Marital status of parents**		
Married	59	17.61
Single	86	25.67
Divorced	159	47.46
Widowed	31	9.25
**Siblings**		
Yes	178	53.13
No	157	46.86

## Data Analysis and Results

### Measurement Model

**Figure 2:**
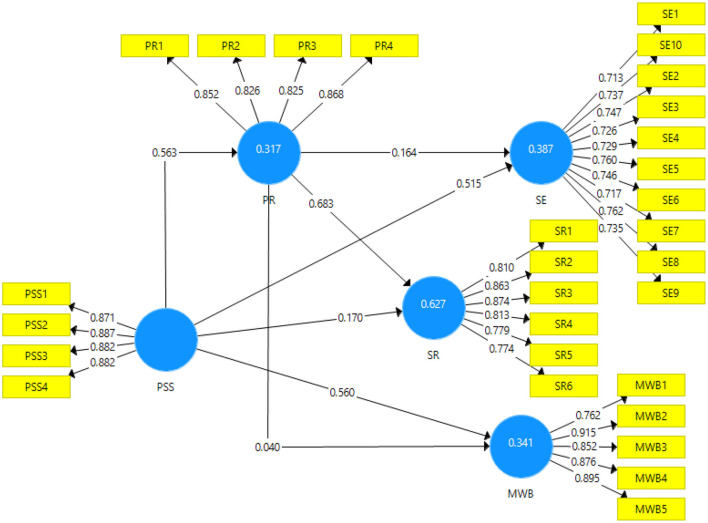
Output of measurement model. PSS, Perceived social support; PR, Psychological resilience; SE, Self-esteem; SR, School readiness; MWB, Mental wellbeing.

Output of the measurement model can be seen in Figure 2.

Table 2 gives a detailed description of the preliminary tests related to reliability and convergent validity of the data. This table shows the factor loadings, variance inflation factor (VIF), average variance extracted (AVE) and composite reliability. According to Dash and Paul (2021), factor loadings of the items should be above 0.7. In this study, all the factor loadings are well above this cut-off value showing the inclusion of the items in the variable. The minimum loading is 0.713 for SE1. The VIF, according to Craney and Surles (2007), should be <5.5 to show acceptable inflation factor in the items. In this study, the maximum VIF noted is 4.0 for the item MWB2 making all items included in their respective scales. The AVE, according to Dash and Paul (2021) should be higher than 0.5; thus, showing a higher amount of variance than the error. In this study, all values are above this cut-off value. The lowest of all is 0.544 for the variable self-esteem. This shows significance of the convergent validity of the scale used. The composite reliability of the scales, according to Grewal et al. (2004) should be above 0.7. In this study, the minimum composite reliability is 0.90 which is for the variable psychological resilience; thus, ensuring the reliability of the data.

**Table 2:** Model assessment (direct model).

**Variables**	**Factor Loadings**		**VIF**	**Composite**	**AVE**
				**reliability**	
Mental wellbeing	MWB1	0.762	1.591		
	MWB2	0.915	4.095	0.935	0.742
	MWB3	0.852	2.658		
	MWB4	0.876	3.371		
	MWB5	0.895	3.844		
Psychological resilience	PR1	0.852	2.159	0.908	0.711
	PR2	0.826	2.038		
	PR3	0.825	1.956		
	PR4	0.868	2.225		
Perceived social support	PSS1	0.871	2.489	0.932	0.775
	PSS2	0.887	2.713		
	PSS3	0.882	2.556		
	PSS4	0.882	2.610		
Self-esteem	SE1	0.713	2.002	0.922	0.544
	SE10	0.737	2.022		
	SE2	0.747	2.812		
	SE3	0.726	2.414		
	SE4	0.729	1.748		
	SE5	0.760	2.913		
	SE6	0.746	2.912		
	SE7	0.717	2.578		
	SE8	0.762	3.446		
	SE9	0.735	2.046		
School readiness	SR1	0.810	2.660	0.925	0.672
	SR2	0.863	2.907		
	SR3	0.874	3.576		
	SR4	0.813	2.451		
	SR5	0.779	2.206		
	SR6	0.774	2.322		

In this study, the discriminant validity is tested with the help of two most commonly used tests, i.e., Fornell and Larcker criterion and heterotrait–monotrait (HTMT) ratio. According to Franke and Sarstedt (2019), the cut-off value for HTMT ratio is 0.9 and in this study, the values of the HTMT ratio obtained are lesser than this value. The maximum value of this discriminant validity checker is 0.646 which is for perceived social support and self-esteem. The results for HTMT ratio are given in Table 3.

**Table 3:** Discriminant validity heterotrait–mono trait ratio.

	**MWB**	**PR**	**PSS**	**SE**	**SR**
MWB					
PR	0.393				
PSS	0.636	0.635			
SE	0.620	0.489	0.646		
SR	0.460	0.874	0.612	0.560	

*PSS, Perceived social support; PR, Psychological resilience; SE, Self-esteem; SR, School readiness; MWB, Mental wellbeing*.

In this study, Table 4 gives the details for the Fornell and Larcker criteria. According to Henseler et al. (2015), the values of each column should show the highest value at the top for data exhibiting the discriminant validity. In this study, the highest value of each column is at the top indicating the acceptance of the discriminant validity of the scales.

**Table 4:** Discriminant validity (Fornell and Larcker criteria).

	**MWB**	**PR**	**PSS**	**SE**	**SR**
MWB	0.861				
PR	0.356	0.843			
PSS	0.583	0.563	0.880		
SE	0.571	0.454	0.607	0.737	
SR	0.423	0.779	0.555	0.522	0.820

*PSS, Perceived social support; PR, Psychological resilience; SE, Self-esteem; SR, School readiness; MWB, Mental wellbeing*.

The *R*^2^-value shows the regression fit of the model. A value more than 50% indicates that the model is substantially good. In this study, the highest value for *R*^2^ is exhibited by the variable school readiness showing 62.7% model fit, followed by the variable self-esteem showing 38.7% model fit. Furthermore, the variable of mental wellbeing has shown 34.1% model fit followed by psychological resilience which is 31.7%. Overall, the model has shown a good model fit with the current variables. The *F*^2^-values indicate change caused in the *R*^2^ when a particular exogenous variable is removed from the model. It is said to be small if it is ≥0.02, medium if ≥0.15, and large if ≥0.35 (Cohen, 1992). In this study, a greater *F*^2^ value has been found for the variables, psychological resilience and school readiness, which is 0.85 followed by the relationship of psychological resilience and perceived social support, which is 0.46. A medium effect has been found for the effect of perceived social support on mental wellbeing which is 0.32 and self-esteem which is 0.29.

### Structural Model

**Figure 3:**
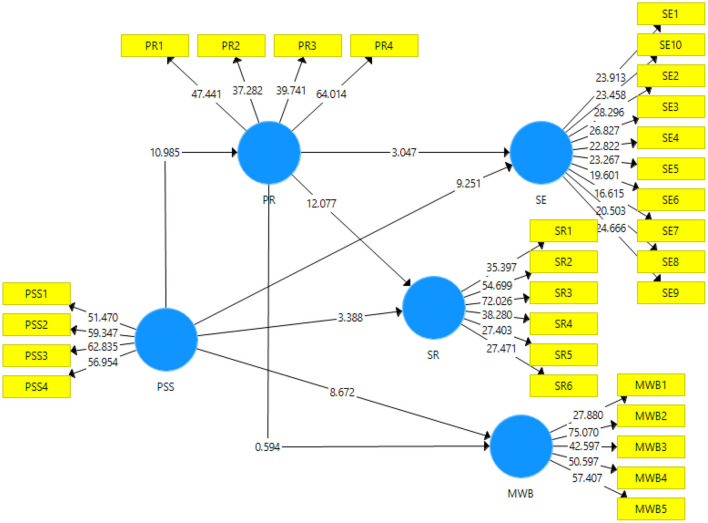
Output of structural model. PSS, Perceived social support; PR, Psychological resilience; SE, Self-esteem; SR, School readiness; MWB, Mental wellbeing.

Output of the structural model can be seen in the Figure 3 below. It is used to test the acceptance or rejection of the hypotheses that have been posited in the literature review using the t-statistics and *p*-value. The effects of independent variables on the dependent variables and the mediating roles have been tested through bootstrapping with 5,000 subsampling at 95% confidence interval.

Table 5 exhibits the direct effects that have been posited in the study. The first direct effect is about the impact of perceived social support on the self-esteem has been accepted with *t*-statistic = 9.25 and *p* = 0; thus, accepting Hypothesis H1. The second direct effect is about the impact of perceived social support on the mental wellbeing has been accepted with *t*-statistics = 8.67 and *p* = 0; thus, accepting Hypothesis H2. The third direct effect is about the impact of perceived social support on the school readiness has been accepted with *t*-statistics = 3.38 and *p* = 0.001; thus, accepting Hypothesis H3. The fourth direct effect is about the impact of perceived social support on the psychological resilience has been accepted with *t*-statistics = 10.98 and *p* = 0; thus accepting Hypothesis H4. The fifth direct effect is about the impact of psychological resilience on self-esteem has been accepted with *t*-statistics = 3.04 and *p* = 0.002; thus, accepting Hypothesis H5. The sixth direct effect is about the impact of psychological resilience on mental wellbeing has been rejected with *t*-statistics = 0.59 and *p* = 0.552; thus, rejecting Hypothesis H6. The seventh direct effect is about the impact of psychological resilience on the school readiness has been accepted with *t*-statistics = 12.07 and *p*-value = 0, thus accepting Hypothesis H7.

**Table 5:** Direct effects of the variable.

**Paths**	**H**	**O**	**M**	**SD**	***t*-Statistics**	***p***	**Results**
PSS → SE	H1	0.515	0.519	0.056	9.251	0.000	Accepted
PSS → MWB	H2	0.560	0.562	0.065	8.672	0.000	Accepted
PSS → SR	H3	0.170	0.175	0.050	3.388	0.001	Accepted
PSS → PR	H4	0.563	0.562	0.051	10.985	0.000	Accepted
PR → SE	H5	0.164	0.166	0.054	3.047	0.002	Accepted
PR → MWB	H6	0.040	0.045	0.068	0.594	0.552	Rejected
PR → SR	H7	0.683	0.683	0.057	12.077	0.000	Accepted

*PSS, Perceived social support; PR, Psychological resilience; SE, Self-esteem; SR, School readiness; MWB, Mental wellbeing*.

Table 6 exhibits the indirect effects that have been posited in the literature review of the study. The first indirect effect is about the mediating role of psychological resilience between the perceived social support and self-esteem has been accepted with *t*-statistics = 2.76 and *p* = 0.006; thus accepting Hypothesis H8. The second indirect effect is about the mediating role of psychological resilience between the perceived social support and mental wellbeing has been rejected with t-statistic = 0.59 and *p* = 0.55, thus rejecting Hypothesis H9. The third indirect effect is about the mediating role of psychological resilience between the perceived social support and school readiness has been accepted with *t*-statistic = 8.09 and *p* = 0; thus accepting Hypothesis H10.

**Table 6:** Indirect effects of the variable.

**Paths**	**H**	**O**	**M**	**SD**	***t*-Statistics**	***p***	**Results**
PSS → PR → SE	H8	0.092	0.094	0.033	2.763	0.006	*Accepted*
PSS → PR → MWB	H9	0.023	0.025	0.039	0.591	0.555	Rejected
PSS → PR → SR	H10	0.385	0.384	0.048	8.093	0.000	*Accepted*

*PSS, Perceived social support; PR, Psychological resilience; SE, Self-esteem; SR, School readiness; MWB, Mental wellbeing*.

## Discussion

The literature showed a gap with regard to the psychological resilience of the LB children which has to be bridged; therefore, the data was collected from the LB children in China. This research intends to examine the impact of perceived social support on mental wellbeing, self-esteem, and school readiness with the mediating role of psychological resilience among the LB children of China. Some direct relationships were examined in the study including the impact of perceived social support and psychological resilience on mental wellbeing, the influence of perceived social support and psychological resilience on self-esteem, the effect of perceived social support and psychological resilience on school readiness, and the role of perceived social support on psychological resilience. Also, the study explored the mediating role of psychological resilience in the relationship between perceived social support and mental wellbeing, between perceived social support and self-esteem, and between perceived social support and school readiness.

Hypothesis H1 of the study has been accepted which posited that perceived social support Hypothesis H1 of the study has been accepted which posited that perceived social support has an effect on self-esteem. Similar findings were found by Fellmeth et al. (2018) who stated that studies have shown that individuals with high social support tend to have higher levels of self-esteem. In addition, according to Gu (2021), perceived social support is closely related to self-esteem. Social support is a crucial factor that enhances the self-esteem of the LB children. Moreover, Hypothesis H2 of the study has been accepted which posited that perceived social support has an effect on mental wellbeing. These results are harmonious with the findings of Wang and Xie (2020) who asserted that studies have shown that perceived social support is positively associated with the mental wellbeing of the LB children because these children need attention and care from others. Wang and Liu (2021) also found that teachers can assess the student's academic performance and provide reasonable suggestions which would help solve psychological problems and promote the mental wellbeing of students.

Hypothesis H3 of the study has been accepted which posited that perceived social support has an effect on school readiness. These findings are synchronous with the findings of Chen et al. (2021) who explained that school readiness considers the competencies of children as the outcome of interest and emphasizes the social system within which the children are embedded. Similarly, Mathis et al. (2022) figured out that the school readiness is developed when the child is mentally prepared to enter school, and it becomes quite challenging for the child to engage in school readiness without their parents. Hypothesis H4 of the study has been accepted which posited that perceived social support has an effect on school readiness. Ran et al. (2022) also found similar results that high social support increases self-confidence decreases the likelihood of engaging in risky behavior, and fosters effective coping strategies (e.g., psychological resilience). Dai and Chu (2018) also revealed that perceived social support also encourages healthy coping behavior and enhances emotional regulations such as anxiety, depression mistrust, and fear. The reason is psychological resilience is developed when children received social support.

Hypothesis H5 of the study has been accepted which posited that psychological resilience has an effect on self-esteem. Similar findings were found in the study conducted by Yu et al. (2022) which showed that psychological resilience reduces depression and anxiety among the LB children while improving their self-esteem and self-efficacy. Likewise, Gabrielli et al. (2022) conducted a study on the psychological resilience of LB children and found that psychological resilience increases self-esteem and reduced depression. However, Hypothesis H6 of the study got rejected which posited that psychological resilience has an effect on mental wellbeing. These results are contrary with the findings of Cui et al. (2021) who claimed that despite threatening circumstances, the LB children can develop mental wellbeing through psychological resilience because resilience enables them to quickly overcome challenging situations. The findings obtained by Parviniannasab et al. (2022) are also contrary to the findings of this study. Parviniannasab et al. (2022) claimed that the children having low psychological resilience can easily develop symptoms of depression, anxiety, and would feel lonely, which would ultimately result in lower mental wellbeing. The possible reason is that psychological resilience is more related to the strength possessed by the children and it is not linked with mental wellbeing of the children. The mental wellbeing is strongly related to social networks. Hypothesis H7 of the study has been accepted which posited that psychological resilience has an effect on school readiness. These results are harmonious with the findings of Fan and Lu (2020) who claimed that the LB children who have a “very weak” to “no relationship” with their parents cannot develop school readiness as they are psychologically empowered. Ramakrishnan and Masten (2019) also claimed that the researchers found that the LB children are reluctant to enter school because of a lack of psychological wellbeing.

Hypothesis H8 of the study has been accepted which posited that psychological resilience acts as mediator in the relationship between perceived social support and self-esteem. Hypothesis H10 of the study has been accepted which posited that psychological resilience mediates the relationship between perceived social support and school readiness. These findings and results were similar to the findings of Zhou et al. (2020) who asserted that resilience among such children allows them to effectively deal with the LB experiences and reduce the negative impact of situations resulting from the lack of social support. Also, the findings were in harmony with the results obtained in the study by Chen et al. (2021) who stated that different types of social support such as neighbor support can positively impact the enhancement of resilience of children. Zhang et al. also stated that psychological resilience is a positive factor that helps the LB children to cope up with adverse situations; therefore, it becomes significant to develop perceived social support to establish psychological resilience in such children. However, Hypothesis H9 of the study got rejected which posited that psychological resilience mediates the relationship between perceived social support and mental wellbeing. These results are contrary with the findings of Chen et al. (2021) who stated that social support from friends and peers can help improve the psychological resilience of children. This is because mental wellbeing is not improved by psychological resilience because the LB children require other factors such as self-efficacy, support from peers, and teachers to improve their mental wellbeing.

### Theoretical Contribution

This study has contributed to filling the gap in literature by fulfilling the objective. First of all, this study has contributed by finding that perceived social support has a significant positive impact on mental wellbeing. It has also been found that perceived social support has a significant positive impact on self-esteem and school readiness. It has further examined that perceived social support has a significant positive impact on psychological resilience. Moreover, this study also contributed to the literature by finding that psychological resilience has a significant and positive impact on self-esteem and school readiness. Another important contribution of the study is that psychological resilience has proved itself as a significant mediating factor in the relationship between perceived social support and self-esteem the study further found that psychological resilience significantly mediates the relationship of perceived social support and school readiness of the LB children.

## Practical Implications

This study has encompassed certain practical implications that will be very important for the educational institutes, boarding organizations, and other grooming institutes to understand how they should tackle the LB children. It is important that the schools and society around takes the responsibility to provide such conducive and favorable environment to the LB children that can help them grow in a better way. First of all, the care and dependability of the LB children are ensured by the parents, teachers, and the caregivers to reinforce and maintain their mental wellbeing. In addition, the relationship of the LB children is ensured with other fellows, so they also help and support them in extracurricular activities. Organizations and the schools should also provide such environment that encourages the social networking of the students making sure that LB children are included. At civil level, a system for social welfare should be introduced that helps the LB children socially, morally, and financially so they can be active and productive participants of the society.

## Limitation and Recommendations

This study has some limitations as well-despite the theoretical importance and practical implications. First of all, this study has taken the population from the Mainland China; however, it is a worldwide issue and this study should be replicated in other parts of the world so as to understand the preference of the schools and the social set up including their social support for the left-behind children. Second, the data has been collected through convenience sampling that may have created a bias in the responses along with social desirability bias that might have affected the respondents about their responses for the survey. Therefore, in the future, a probability sampled study is recommended to avoid such biases in the study if occurred. Third, it is recommended that other variables (self-efficacy, children club, etc.) that might affect the self-esteem, mental wellbeing, and school readiness of the LB children. Further, it is also recommended to introduce the moderation in the current model so to get better insight into this study.

## Conclusion

The LB children is the on-going development of urban areas and the difference in the social system of urban and rural areas which provides various opportunities for people to move to urban regions of better income (Li et al., 2021). Additionally, it had also been suggested to incorporating other comprehensive measures that could affect the LB children; therefore, this study aimed to examine the effect of perceived social support on school readiness, mental wellbeing, and self-esteem with the mediation of psychological resilience among the Chinese LB children. In this regard, this study has investigated the impact of perceived social support on the self-esteem, mental wellbeing, and the school readiness of the LB children and it found a positive impact of perceived social support on self-esteem development, mental wellbeing, and the school readiness of left being children. For this purpose, the school-going LB children aging between 8 and 12 years has been taken as the population frame for this study. The study has further investigated the impact of perceived social support on the psychological resilience of the LB children. The study further investigated the impact of psychological resilience on the self-esteem, mental wellbeing and school readiness. The study has also investigated the mediating role of psychological resilience between perceived social support as independent variable and self-esteem, mental wellbeing, and school readiness as the dependent variables. The findings of the study give the schools and the society around important insights to take the responsibility to provide such conducive and favorable environment to the LB children that can help them grow in a better way.

## Data Availability Statement

The original contributions presented in the study are included in the article/supplementary material, further inquiries can be directed to the corresponding author.

## Author Contributions

The author confirms being the sole contributor of this work and has approved it for publication.

## Conflict of Interest

The author declares that the research was conducted in the absence of any commercial or financial relationships that could be construed as a potential conflict of interest.

## Publisher's Note

All claims expressed in this article are solely those of the authors and do not necessarily represent those of their affiliated organizations, or those of the publisher, the editors and the reviewers. Any product that may be evaluated in this article, or claim that may be made by its manufacturer, is not guaranteed or endorsed by the publisher.
